# Editorial: The role of RNA regulation in cancer pathogenesis and therapeutic strategies

**DOI:** 10.3389/fonc.2026.2008301

**Published:** 2026-10-07

**Authors:** Senn Wakahashi, Maho Azumi, Kentaro Minagawa

**Affiliations:** 1 Department of Gynecology/Obstetrics, Kobe University Hospital, Kobe, Japan; 2 Department of Hematology/Oncology, Penn State Cancer Institute, Hershey, PA, United States; 3 Department of General Medicine, Kobe University Hospital, Kobe, Japan

**Keywords:** MCPIP, post-transcripional control, regnase, RNA-binding proteins, TFL

Post-transcriptional regulation of gene expression is a critical determinant of inflammatory protein production and cancer biology. Although transcription initiates cellular responses, the stability, localization, processing, and translation of RNA ultimately determine the magnitude and duration of protein expression. More than 1,500 RNA-binding proteins (RBPs) participate in these processes, highlighting the complexity of RNA-mediated regulation. Among these, CCCH-type zinc-finger proteins—including tristetraprolin (1, 2), Roquin (3), and the Regnase family (4, 5)—play important roles in controlling inflammatory and immune responses.

Inflammatory cytokine mRNAs are often intrinsically unstable, allowing rapid induction and subsequent resolution of inflammatory responses. Regulatory sequences within the 3′ untranslated regions, including AU-rich elements, microRNA-binding sites, and stem-loop structures, provide recognition sites for RBPs and RNA-silencing machinery. Through these mechanisms, inflammatory transcripts such as IL-6, TNF-α, IL-2, and IL-17A can be rapidly degraded, or their translation modulated. This post-transcriptional checkpoint is essential for preventing excessive or persistent inflammation.

TFL (ZC3H12D/Regnase-4/MCPIP-4), a member of the ZC3H12 family, contains a CCCH-type zinc-finger domain involved in RNA recognition and a PIN-like RNase domain involved in RNA degradation (6). Although it shares structural and functional features with Regnase-1 (ZC3H12A), the Regnase family members have distinct expression patterns and RNA targets (7). TFL regulates inflammatory transcripts and appears to function as a negative regulator of immune activation. Its localization to P-body-associated RNA granules further suggests that it participates in dynamic networks controlling RNA storage, degradation, and translation.

The immunological significance of TFL is particularly evident in chronic inflammation and autoimmunity. Experimental autoimmune encephalomyelitis demonstrates that loss of TFL can enhance inflammatory responses and pathogenic Th17-cell accumulation, suggesting an important role in controlling persistent inflammation and disease resolution (8). Regulation of TFL by transcription factors such as BHLHE40 may further connect RNA metabolism with inflammatory T-cell programs. Because BHLHE40-positive and GM-CSF-producing T cells are implicated in graft-versus-host disease (GVHD) (9), TFL represents a potentially important but still incompletely characterized regulator of GVHD. Direct experimental evidence, however, is needed to establish this connection.

TFL also influences cancer biology. Reduced TFL expression has been associated with lymphoma and other malignancies, while experimental loss of TFL can enhance cellular proliferation, NF-κB signaling, inflammatory cytokine production, and tumor-associated inflammation (5, 10). These observations suggest that TFL may function not simply as a tumor suppressor within malignant cells but also as a regulator of the tumor microenvironment and systemic inflammatory consequences of cancer. Its potential effects on NK cells and other immune populations further raise the possibility that RNA-regulatory pathways may influence antitumor immunity. Interestingly, RNA-regulatory proteins are not limited to their function within RNA deregulation. TFL was even found on the cell surface as a receptor for extracellular mRNA, including IL-1β. Tumor stimulation alters the localization of TFL in immune cells. In a mouse model, TFL is present on the cell surface, whereas tumor exposure causes TFL to rapidly move into the intracellular compartment within a few hours (11), contributing to enhanced immune activity against cancer.

TFL functions as an RNA-binding protein and was reported to bind the 3′-UTR of IL-1β mRNA, facilitating its movement into the nucleus. This process was associated with increased anti-apoptotic activity, NK-cell mobility, and IFN-γ production, potentially enhancing NK-cell–mediated antitumor and antimetastatic effects.

The articles included in this Research Topic demonstrate that RNA regulation operates at multiple interconnected levels. Xie et al. showed that acetyl tributyl citrate may affect prostate cancer through epigenetic regulators such as EZH2, DNMT1, and PRMT5. Tomita et al. highlighted the therapeutic potential of synthetic short RNAs. Zhang et al. reviewed miR-17 as an oncogenic microRNA involved in proliferation, epithelial–mesenchymal transition, invasion, angiogenesis, and treatment resistance. Molefi et al. demonstrated the importance of abnormal transcriptional regulation, RNA processing, alternative splicing, and MAPK signaling in aggressive endometrial cancer.

Other contributions illustrate additional mechanisms. Shen et al. described the lncRNA ADAMTS9-AS2 as an epigenetic regulator that interacts with DNMT3B and prevents methylation-mediated silencing of CADM2. Sun et al. demonstrated that circNF1 functions as an RNA scaffold for IGF2BP1 and promotes stabilization of HMGA2 mRNA, illustrating how circular RNAs can regulate gene expression through RNA–protein interactions. Yang et al. further emphasized the broader importance of circRNA–protein interactions in cancer. Wang et al. highlighted epitranscriptomic regulation through METTL1-mediated m7G modification of tRNA, demonstrating how RNA modifications can selectively alter translation and promote cancer progression.

Collectively, these papers illustrate a continuum of RNA regulation extending from chromatin and transcriptional control to RNA processing, mRNA stability, RNA–protein interactions, RNA modification, and translation. These mechanisms are not isolated; rather, they form interconnected regulatory networks that influence tumor proliferation, metastasis, inflammation, immune evasion, and therapeutic resistance.

Understanding these networks may provide new opportunities for precision oncology. RNA-binding proteins, non-coding RNAs, RNA modifications, and RNA-processing pathways may serve not only as biomarkers but also as therapeutic targets. In particular, manipulating RNA-regulatory pathways could have complementary effects on both malignant cells and the immune microenvironment. Future studies should define the direct RNA targets and context-dependent functions of those RNA regulatory proteins, including TFL and other Regnase families, clarify their roles in autoimmune disease, including GVHD, as well as cancer, and determine whether these pathways can be therapeutically exploited to enhance cancer immunotherapy.

As the editorial team, we hope that this Research Topic will stimulate further investigation into the expanding field of RNA regulation and contribute to the development of innovative diagnostic and therapeutic strategies for cancer and inflammatory disease.
